# Continental drift? Do European clinical genetic testing laboratories have a patent problem?

**DOI:** 10.1038/s41431-019-0368-7

**Published:** 2019-03-07

**Authors:** Johnathon Liddicoat, Kathleen Liddell, Arlie H. McCarthy, Stuart Hogarth, Mateo Aboy, Dianne Nicol, Simon Patton, Michael M. Hopkins

**Affiliations:** 1 Faculty of Law, Centre for Law, Medicine and Life Sciences, University of Cambridge, Cambridge, United Kingdom; 2 Department of Zoology, University of Cambridge, Cambridge, United Kingdom; 3 Department of Sociology, University of Cambridge, Cambridge, United Kingdom; 4 Faculty of Law, Centre for Law and Genetics, University of Tasmania, Hobart, Australia; 5 European Molecular Genetics Quality Network, Manchester, United Kingdom; 6 Science Policy Research Unit, University of Sussex, Brighton, United Kingdom

**Keywords:** Genetic testing, Diagnostic markers, Social sciences

## Abstract

Recent US Supreme Court decisions have invalidated patent claims on isolated genomic DNA, and testing methods that applied medical correlations using conventional techniques. As a consequence, US genetic testing laboratories have a relatively low risk of infringing patents on naturally occurring DNA or methods for detecting genomic variants. In Europe, however, such claims remain patentable, and European laboratories risk infringing them. We report the results from a survey that collected data on the impact of patents on European genetic testing laboratories. The results indicate that the proportion of European laboratories that have refrained from providing associated testing services owing to patent protection has increased over the last decade (up from 7% in 2008 to 15% in 2017), and that the non-profit sector was particularly strongly affected (up from 4% in 2008 to 14% in 2017). We renew calls for more readily available legal support to help public sector laboratories deal with patent issues, but we do not recommend aligning European law with US law at present. Watchful monitoring is also recommended to ensure that patents do not become a greater hindrance for clinical genetic testing laboratories.

## Introduction

The patentability of genetic inventions and discoveries has been socially, legally and ethically controversial for decades [1, 2]. In the 1990s, surges in the number of patents claiming gene-related inventions [3]—especially isolated gDNA—prompted investigations around the world to assess whether patents had adversely impacted genetic testing services [4–9]. In the United States, some studies showed negative impacts. For example, in a 2001 survey, Cho et al. [10] found that 53% of US laboratories had decided not to develop or perform a test (either for research or clinical purposes) due to a patent. Furthermore, in 2010, a US government report found that patents had generally limited the availability of services rather than promoted them [11]. Other US studies reached different conclusions, including that patents rarely hamper academic and commercial research [12, 13], and that there is positive correlation between DNA-based patents and investment in R&D [14].

The impediments reported in these surveys, as well as others, motivated the American Civil Liberties Union and the Association of Molecular Pathology to challenge patenting of isolated DNA in the United States [15]. The Supreme Court responded sympathetically. In *Association for Molecular Pathology v. Myriad Genetics Inc* [16] (*Myriad*), the Court invalidated patent claims to isolated gDNA on the grounds that they were not ‘markedly different’ to what exists in nature. One year earlier in unrelated litigation, the Court in *Mayo Collaborative Services v. Prometheus Laboratories Inc* (*Mayo*) held that the mere application of natural phenomena using conventional techniques was not patentable either. The patent in *Mayo* concerned a method for more precisely dosing a drug based on observing the patient’s drug-metabolite levels. However, courts have since applied the case to invalidate a variety of patents relevant to methods for diagnosing conditions based on observing genetic characteristics, including methods for non-invasive prenatal testing (NIPT) [17] and for identifying whether a patient has a disease-linked variant [18]. That said, some key aspects of genetic testing still remain eligible for US patent protection; including laboratory-engineered DNA (e.g., complementary DNA, and recombinant DNA) [19] and sequencing techniques [20].

The patent law changes in the United States have not (yet) been mirrored in European patent law, and are currently the focus of intense enquiry and discussion in the United States [21–23]. Should we implement similar law reform in Europe? What impact are gene-related patents having on European clinical genetic laboratories? Is the impact as significant as it was in the United States prior to the US Supreme Court decisions? These questions were the motivation for this study.

## Background

European patent law provides relatively broad protection for gene-related patents. The European Directive on the Protection of Biotechnological Inventions (98/44/EC) states that isolated DNA sequences shall be patentable in European Member States. The European Patent Convention is interpreted in the same way by the European Patent Office. The inherent patentability of isolated DNA sequences was confirmed in the European BRCA litigation [24]. The patents were reduced substantially in scope but not rejected for being unpatentable subject matter [25, 26]. Genetic diagnostic methods are also patent eligible in Europe, even when the techniques implementing the methods are conventional. For example, a UK court recently held that, in contrast to the US case, the European patent for NIPT is valid [27]; and the apex patent court in Germany, the Bundesgerichtshof, recently confirmed the validity of patent claims on both the isolated human FLT3 gene and methods for evaluating whether a patient has a certain variant in FLT3 [28, 29]. Although the European Patent Convention excludes methods of diagnosis on the human body, this exclusion has been interpreted narrowly and does not exclude in vitro diagnostic methods such as methods of genetic testing based on blood, cells or saliva samples [30, 31].

It is ~ 10 years since European laboratories were last surveyed about the impact of gene-related patents. At the time, patents were not having as much of an impact on European laboratories as they were in the United States. Gaisser et al. [5] found in 2008 that only 7% of European laboratories reported discontinuing a test due to a patent. This and other results led the authors to conclude that there was no patent ‘menace in Europe—yet’. Stressing the time-limited nature of their conclusion, however, the authors suggested that patents might affect services adversely in the future, especially as new technology might increase both the number of people and genes tested. A recent review of the impact of patents on medical practice in the UK concluded that ‘neither current nor future medical practices appear to be impinged by gene patents’ [32], but this review was not based on any new data.

In the decade since Gaisser et al.’s survey, there have been significant developments. Accordingly, a fresh survey is essential if we are to understand the present-day impact of gene-related patents on European clinical genetic laboratories. For instance, the power and scale of sequencing and testing techniques have rapidly advanced since 2008—especially with the uptake of whole genome/exome sequencing. Governments around the world have cumulatively committed vast sums to genomic medicine programmes, with a primary aim of stratifying patients based on genetic traits to achieve more accurate diagnosis, prognosis and treatment [33]. With these technological and financial inputs, the Western European clinical molecular genetic testing ‘industry’ has grown markedly—a 2015 commercial report found that the industry has been growing at over 10% since 2013 and predicted it to be worth US$3.08 billion by 2020 [34]. At the same time, it is possible that the US cases have influenced companies to shift the focus of patent enforcement actions from the US to Europe, where they, arguably, stand better chances of success [35].

This survey-based study seeks a present-day understanding of the scale of patent-based impacts on clinical genetic testing services. In order that one can see change over time in Europe, as well as inter-regional comparison (with the US and Australia), this study follows earlier surveys in covering: laboratories’ awareness of patent rights; whether patentees have enforced their rights; how laboratories have responded to enforcement efforts; whether laboratories have obtained patent licenses either bundled with commercial testing kits or through contractual licenses; whether laboratories have outsourced tests in an attempt to avoid patent infringement; whether patents have restricted laboratories’ R&D; and whether laboratories have refrained from performing a test due to a patent. These questions do not definitively answer whether patients are able to access specific clinical genetic services. However, they do indicate the nature and scale of patent-based impacts on laboratories.

## Methods

### Survey instrument and design

The survey was based on those used in Gaisser et al.’s 2008 European survey and a similar Australian study by Nicol et al. [6] in 2012. A pilot survey was tested by designated persons in the research team, and two independent persons respectively specialising in genetics policy for the UK civil service and a clinical genetics. Participation was voluntary and estimated to take 20 minutes. Laboratories were not required to answer every question. The survey was provided in English only. The survey referred to gene-related impacts of patents on clinical genetic testing laboratories. This language deliberately left open the possibility of laboratories raising issues with patents on nucleic acid biomarkers, methods of detection and diagnosis, and platform technologies. There were several reasons for taking this wide approach rather than, for example, limiting the study to biomarker patents: laboratories are affected by patents relating to biomarkers, methods, and platforms; US legal developments have affected all such patents; and there is no strict demarcation in the sense that platform technologies can involve a variety of nucleic acid markers and methods of clinical diagnosis.

### Distribution

The survey was distributed via two channels. First, on 8 May 2017, it was emailed to 1068 European molecular genetics laboratories listed on a database provided by Orpha.net (we cleaned and updated some contacts in the database but no large changes were made). Second, on 5 June 2017, the survey was sent to the 561 European member laboratories of the European Molecular Genetics Quality Network (EMQN). Two email reminders were sent via each channel. Owing to possible cross-posting on the two channels, respondents were asked to ensure their laboratory had not already completed the survey.

Gaisser et al. used the EuroGentest laboratory network to distribute their survey in 2008. The list of genetic laboratories in that network was incorporated into Orpha.net in 2008. The reason for using a second channel in this study (EMQN) was that 101 emails sent to addresses listed on the Orpha.net database bounced, raising the possibility that many more emails were not received, likely owing to staff turnover.

The number of laboratories contacted successfully via the two channels was estimated by a cohort of follow-up telephone calls after the survey closed on 6 July 2017. Through the telephone calls, we estimated that the survey was received by 806 laboratories (see Supplementary Material). By comparison, the 2008 European survey was emailed to 289 laboratories.

Two hundred and four laboratories that provide human clinical molecular genetic testing started the survey, and 158 completed it; meaning that 25% began it and 20% completed it. By comparison, the 2008 European survey had 83 responses, and the 2001 US survey had 132 completed responses. The 2008 European survey is the only study to have assessed response bias; it found no response bias in terms of laboratory size, nationality or reason for not responding.

### Statistical analysis

The number of complete responses (158) makes this the largest study on this topic. It was large enough to compare the results of this survey with Gaisser et al.’s 2008 European survey with 90% confidence intervals (CIs). These calculations are located in the Supplementary Material. The sample size was too small for robust statistical analysis between sub-groups in the sample. For example, we could not say with confidence or statistical significance that for-profit laboratories (one sub-group) were more likely to discontinue clinical genetic tests due to a patent than publicly funded genetic laboratories (another sub-group). Nevertheless, we follow the earlier survey studies and report associations between sub-groups based on descriptive statistics.

## Results and discussion

### Laboratories’ characteristics

The number of laboratories emailed in this study (~ 806) compared with the number emailed in the 2008 study (289) suggests that there has been a substantial growth in the European clinical genetic sector. In part, this increase might be driven by the increased visibility of laboratories, which in turn is due to better web-based networking. The results also show that 141 (75%) laboratories are part of non-profit organisations, and 47 (25%) are part of for-profit organisations. In contrast, the 2008 survey found that only 7% of laboratories were from for-profit organisations, an 18% difference from the 2018 findings (90% CI = ± 8%). This increase in the for-profit sector fits the data reported above about the financial growth in the industry. Laboratories, regardless of whether they identified as profit or non-profit, were also asked to specify what type of organisation they were from. Government-funded hospitals, universities, and small- or medium-size companies together constituted 76% of all respondents (Table 1).

**Table 1:** Organisations to which laboratories belong

Type of organisation	For profit	Non profit	Total for organsiation type	% of all laboratories
Blood bank	0	2	2	1%
Private hospital	3	0	3	2%
Company–large	6	0	6	3%
Public research institute	0	10	10	5%
Non-profit/non-state hospital	0	12	12	6%
Private research institute	5	7	12	6%
Company–SME	27	2	29	16%
University	4	43	47	25%
Government-funded hospital	1	65	66	35%
Total	46	141	187	100%

Geographically, 29 (15%) laboratories werre based in Italy, 24 (12%) in Germany, 23 (12%) in Spain, 19 (10%) in the UK, 17 (9%) in France, and another 78 (41%) were from 20 different countries.

The size of laboratories was gauged by asking how many test reports they issued per year: 11 (9%) issued < 100; 39 (21%) issued 100–499; 23 (12%) issued 500–999; 77 (41%) issued 1000–5000; and 39 (21%) issued > 5000. The majority of responding laboratories were thus relatively large.

The same question was asked in the 2008 survey. Comparatively, the proportion of laboratories in the three middle size categories are similar, but the proportion of the smallest and largest laboratories are noticeably different. From 2008 to 2017, the proportion of laboratories providing < 100 has decreased by 5% (90% CI = ± 7%), whereas the proportion providing > 5000 tests has increased by 10% (90% CI = ± 9%) (Supplementary Material).

Laboratories also responded to questions about types of testing, whether laboratory staff have been involved in patenting, and respondents’ roles in the laboratories (Supplementary Material).

### Awareness of patent rights

Laboratories were asked whether they conducted tests using patented genes or methods of diagnosis: 70 (37%) stated they did, whereas 63 (34%) said they did not, and 55 (29%) did not know. Ten of these 70 laboratories were from Italy, 9 each from Germany and Spain, 7 each from France and the UK, with an additional 27 laboratories distributed across 13 further countries.

The 2008 European survey asked a slightly different question: whether laboratories conducted tests on patented genes. In response, 18 (22%) laboratories reported they did, 45 (54%) said they did not, and 20 (24%) did not know. Patents on genes often include methods for analysing the gene of interest, thus we suspect these two questions have been interpreted the same way in both surveys. Assuming this is correct, 2008–2017 saw a 16% (90% CI = ± 10%) increase in laboratories knowingly conducting patented tests. What is driving this increase is currently unclear but may be a combination of factors. An obvious factor is that a higher proportion of laboratories are now contacted about potential infringement (see below), but laboratories may simply be more aware of patent rights owing to media coverage of recent European litigation. A greater proportion of laboratories might be infringing patented tests, but this possibility cannot be drawn convincingly from the data.

### Enforcement of patents

Laboratories were asked whether they had been contacted about alleged patent infringement: 21 (13%) stated that they had been, 126 (76%) said they had not been, and 18 (11%) did not know if they had been. This question was not asked in the 2008 European survey, however, a 2010 interview-based study of UK laboratories found that patentees had not enforced any patents against the laboratories interviewed [36], indicating contact from patent owners has increased over time.

This interpretation is reinforced by the following finding. In the present study, 20 of the 21 laboratories that had been contacted disclosed their country: six were from the UK; three from Germany; two each from Belgium, Switzerland, Austria and Sweden; and one each from Italy, France and the Netherlands. This means that 100% (2/2) of the Swedish, 40% (2/5) of the Belgian and 32% (6/19) of the UK laboratories in this survey had been contacted about infringement by patent holders.

Laboratories were asked to list the tests affected by the allegation of patent infringement. Nine laboratories reported one test each, whereas another four reported two tests. Totalled together: 11 listed FLT3, 3 NIPT, 1 RAD51C, 1 Ig/TCR clonality testing and 1 JAK2.

We observed positive associations between laboratories that have been contacted about alleged patent infringement and those that: (a) are larger in size; (b) offer tests on cancer or blood disorders; (c) knowingly undertake tests on patented genes and methods; (d) are from for-profit organisations; and (e) have higher levels of IP support. (Supplementary Material).

Although we observed a positive association between laboratories contacted by patent holders and laboratories that knowingly use patented genes or methods, only 14 of the laboratories that knowingly used gene-related patents had been contacted regarding infringement (25% of the laboratories that answered both questions). This suggests that the majority of laboratories learn they are using patented genes and methods from sources other than infringement allegations. The 2010 interview-based UK study concluded that laboratories deliberately ignored the possibility of patent enforcement [38]. The results of this survey suggest that now, at least for a small number of tests, fewer laboratories ignore the possibility of enforcement and more now choose to respect IP rights. It is not clear what is driving this change in behaviour, but informal clinician feedback suggests that it is due to the risk of enforcement increasing [37]. That is, news of parties enforcing patents has spread throughout the sector.

### Responses to patent protection

Laboratories have several options when faced with a patented genes or methods of diagnosis they do not have authority to conduct. They can (1) choose to not offer the test; and (2) refrain from R&D involving the patented gene or method. Alternatively, licences to use patented genes or methods of diagnosis can be secured in one of two typical ways: (3) by purchasing a commercially supplied testing kit (where the cost of a patent license is bundled into the kit price); and/or (4) by negotiating a patent license to perform the testing in-house, including as a laboratory developed test (LDT) or ‘homebrew’ test (an “unbundled license”). It is less well known that: (5) outsourcing a genetic test can be a way to avoid patent infringement, particularly if the test is outsourced to a country where there is no relevant patent protection [38, 39].

Each of these five responses can have implications for service quality. Obviously, not offering a genetic test limits the availability of the test; less obviously, it limits opportunities for second opinions. Outsourcing can result in longer test turn-around times. A commercial kit may be more expensive for payors and, potentially, offer a restricted analysis to the patient compared with an LDT. On the other hand, a kit may have met more rigorous regulatory checks than the LDT option, but this depends on the regulatory environment [40]. A decision to refrain from R&D makes improvements less likely.

Prior to our survey, it was not known to what extent genetic laboratories were responding to patents that might interfere with their services. The questions were designed to identify whether patent issues underpinned these choices and the relative importance of other issues.

### Genetic test services blocked by patents

**Fig. 1:**
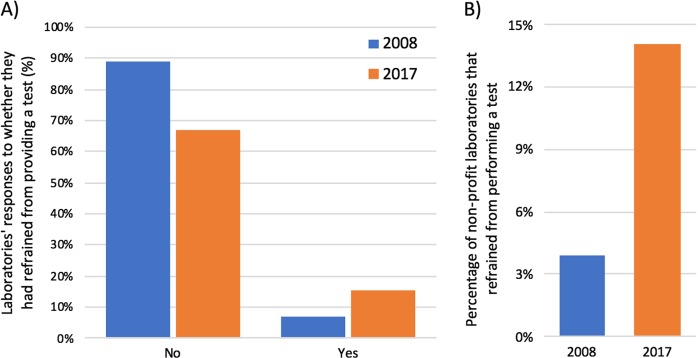
**A** Laboratories’ responses to whether they had refrained from performing test (2008 vs 2017). Compared with the 2008 European survey, the percentage of laboratories that have refrained from performing a test has increased from 7 to 15% (“do not know” responses are omitted). **B** Percentage of non-profit laboratories that chose not to provide a test (2008 vs 2017). Compared with the 2008 European survey, the percentage of non-profit laboratories that chose not to provide a test has increased from 4 to 14%

Laboratories were asked whether they had chosen not to perform a test owing to a patent (i.e., refrained from performing a test). Twenty-six (15%) laboratories reported they had refrained from performing a test owing to a patent, whereas 113 (67%) said patents had not stopped them from performing a test and 30 (18%) did not know if a patent had stopped them from performing a test (Figure 1a). Of the 26 laboratories to refrain from offering a test, 18 (69%) were from non-profit organisations and 8 (31%) from for-profit organisations. Geographically, five laboratories that chose not to provide a test were located in Germany, four each in the UK and Switzerland, three in France, and a further eight laboratories in nine different countries. This means that 36% of Swiss laboratories and 21% of UK laboratories that responded to this survey chose not to perform a test owing to a patent.

Compared with previous surveys, this result indicates that an increased number of European laboratories have refrained from performing a test owing to a patent. The 2008 European survey found that 7% of laboratories had refrained from performing a test, meaning there has been an 8% increase in almost a decade (90% CI = ± 7%). The 2008 study also found that only 4% of laboratories from non-profit organisations had refrained from offering a test, whereas this survey has found this proportion has more than tripled to 14%, a 10% increase (90% CI = ± 7) (Fig. 1b).

The current percentage of affected European laboratories across the sector (15%) is also greater than that found in Australia in 2012 (7%), but markedly lower than the percentages found in the United States in 2001 (25% ceased performing a test after receiving an infringement notification; and 53% did not develop or perform a test for clinical or research purposes owing to a patent.

The laboratories that chose not to perform a test were asked to specify the test in question, and 14 laboratories responded with one test each: 6 listed FLT3; 4 NIPT; 1 ABCB1; 1 RAD51C/CHK2; and 1 chromosome X linkage analysis. An additional laboratory stated they chose not to provide a test for predisposition to cancer but did not provide any specific details.

Previous surveys listed different tests that were not offered owing to a patent (for example, BRCA1/2, TCF7L2). Two reasons for the list changing is that patents will have expired in the intervening time, and more recently patented tests will have been developed. This shows that the expiry of patents does not solve the general issue of patents impacting genetic laboratories.

Laboratories that chose not to provide a test owing to a patent were asked to rate five reasons why a patent might be influential, including among others that the patent involved unreasonable costs, risks of patent enforcement and turn-around time due to insistence that the test be performed elsewhere (e.g., in the patent owner’s laboratory). Respondents rated these reasons on a scale from being irrelevant through to being the deciding factor. All six laboratories that chose not to provide FLT3 testing answered this question, but only three of the four laboratories that chose not to provide NIPT answered. The most prominent reasons to not provide FLT3 were: unreasonable licensing price and risk of patent enforcement. The most prominent reasons to not provide NIPT were: unreasonable licensing cost, the inability to obtain a reagent from a third party, and risk of patent enforcement (see Supplementary Material).

A positive association was found between laboratories that chose not to provide a test owing to a patent and those that: (a) were from for-profit organisations; (b) had higher levels of IP support; (c) received allegations of patent infringement; and (d) conducted tests on patented genes or methods (Supplementary Material).

Although we observed a positive association between laboratories that chose not to provide a test owing to a patent and those that had been contacted about alleged infringement, 11 chose not to offer a test without being contacted (48% of the laboratories to answer both questions). Thus, this result indicates that almost half of the laboratories that chose not to offer a test owing to a patent decided to do so without infringement being alleged. This reinforces points made above about European laboratories being more aware of patents than in previous studies.

### Patents and R&D

One hundred and six (65%) laboratories reported that they conducted R&D, whereas 35 (21%) said they did not and 23 (14%) said they did not know. Research-active laboratories were then asked if they intended to apply (as an inventor) for a patent in the future: 20 (19%) reported they did; 36 (34%) said probably not; 20 (19%) said no and 29 (28%) said they had not thought about it.

Laboratories that did conduct R&D were also asked if they had decided not to conduct R&D involving a genetic test owing to a patent: seven (7%) said they had decided not to conduct R&D owing to a patent, whereas 83 (80%) said patents had not affected their R&D decisions and 14 (13%) did not know. Two laboratories reported the genetic test where they had refrained from R&D. One cited fragile X syndrome; and another cited the ‘Roche diagnostic panel for oncology’.

In comparison, the 2008 European survey found a similar number of research-active laboratories (70%), and a similar number that had decided not to conduct R&D on a test owing to a patent (4%). As previously noted, Cho et al. [10] found that 53% of US laboratories had decided not to develop or perform a test for either clinical or research purposes due to patent (it is unclear from this combined percentage precisely what percentage stopped R&D).

### Kits and bundled licenses

One hundred and sixty-four (88%) laboratories reported that they used commercially-supplied kits, and 23 (12%) said they did not. Laboratories that used kits were then asked if a patent license (or royalty) was included in the purchase price of their kit: 20 (14%) reported that one was included, 12 (8%) said no license was included, and 115 (78%) laboratories said they did not know (see Supplementary Material for which kits were reported as including a license). By contrast, the 2008 European survey found that 4% of laboratories purchased kits that included patent licenses, indicating an increase of 10% (90% CI = ± 7%). A positive association exists between laboratories that purchase kits with bundled licenses and those that undertake tests on patented genes or methods of diagnosis.

Laboratories that used kits were then presented with seven reasons why a laboratory might choose a kit in preference to an LDT and asked to rate each of them on a six-point Likert scale (from very important to very unimportant, including not relevant). We created weighted average ratings for each reason (Table 2). These averages were calculated by creating a weighted count for each reason, and then dividing this count by the number of respondents that rated the reason. The ratings were weighted as follows: very important = 2; important = 1; neutral = 0; unimportant = −1; very unimportant = −2; and not relevant = 0.

**Table 2:** Reasons to use a kit instead of an LDT

Reasons	*n*	Weighted average rating
Saves time	145	1.1
Increased accuracy/confidence	147	1.1
Saves money	143	0.7
Minimises the need for validation per EU/national regulations	147	0.7
Difficulty in performing an LDT	147	0.7
Not having to outsource	144	0.6
Risk of patent enforcement	142	0.1

Weighted average ratings showing that the risk of patent enforcement does not generally influence laboratories to use kits

Saving time and increased accuracy/confidence were the most influential reasons to choose a kit instead of performing an LDT, both have weighted average weights of 1.1 (important). In contrast, risk of patent enforcement was the least important reason, with a weighted average rating of .1 (neutral or not applicable). This reason did, however, obtain a wide range of responses: 7 laboratories (5%) rated it as very important, 32 (23%) as important, 56 (39%) as a neutral consideration, 14 (10%) as unimportant, 6 (4%) as very unimportant (8%) and 27 (19%) as not a relevant consideration. No similar question was asked in the 2008 European survey.

It is clear from these findings that the vast majority of laboratories are choosing to use kits for reasons other than as a response to potential patent infringement. Nevertheless, the finding that 14% of laboratories use a kit that includes a license and 28% of laboratories said that patent enforcement is an important or very important consideration when choosing to use kits shows that many laboratories are conscious of the issue and a non-trivial proportion act on it.

A positive association exists between laboratories from for-profit organisations and those that said patent enforcement was a very important or an important consideration (Supplementary Material).

### Unbundled licenses

Laboratories were then asked if they paid a license fee other than one included in the purchase price of a kit. Nine (5%) laboratories reported they did, whereas 121 (72%) said they did not, and 39 (23%) did not know if they did. Three of the nine that paid a license had been contacted by patent holders regarding patent infringement. It is possible that this contact motivated these three laboratories to take out a license and pay license fees but the results show that it is more common (as seen in six out of the nine laboratories) to pay license fees outside of the purchase price of kits without being contacted about alleged patent infringement. This is a further evidence that not all labs are ignoring patents.

Four laboratories specified which tests they paid licenses for: two listed NIPT and the other two listed next generation sequencing (NGS). As NGS kits typically include a license in the purchase price of the kit, it is unclear whether the laboratories who mentioned it answered the question correctly. One of the laboratories that licensed NIPT was a large, for-profit company in Switzerland, and the other was part of a large, government-funded hospital in the UK. Both said that the license fee was unreasonable. One laboratory listed the price at $70 USD(~€60) per test.

The 5% of laboratories paying a stand-alone license fee is an increase over the 2008 European survey in which only 1% responded positively to this question [10], a difference of 4% (90% CI = ± 4%). This result, however, is lower than the 13% found in the Australian survey and the 27% in the United States survey (although, the US survey did not distinguish between license fees bundled with kits or not).

Respondents were also asked whether they performed any centrally-licensed tests (e.g. tests in which a government negotiated licenses for state hospitals). One hundred and twelve laboratories (66%) reported that they did not perform any centrally licensed test, whereas 55 (32%) said they did not know if they did.

### Patents and outsourcing

Laboratories were asked whether they outsourced testing and their reasons for doing so. Eighty-seven laboratories (52%) stated they did outsource tests, whereas 79 (48%) said they did not. Laboratories that did outsource were then presented with six reasons why a laboratory might choose to outsource and were asked to rate each of them on a six-point Likert scale. Table 3 shows a weighted average rating for each reason (created using the method in Table 2).

**Table 3:** Reasons to outsource tests

Reasons	*n*	Weighted average rating
Low test volume	84	1.08
Saves time	83	0.94
Difficulty in performing test	86	0.91
Saves money	84	0.85
Increased confidence in the test	83	0.64
Risk of patent infringement	81	−0.01

Weighted average ratings showing that the risk of patent enforcement does not generally influence laboratories to outsource tests

The most influential reasons to outsource were: low test volume, saves time and difficulty in performing test. In contrast, risk of patent infringement was rated as the least influential reason: two laboratories (3%) selected it as very important and 12 (15%) as an important consideration. These 14 laboratories were asked which tests they outsourced. Only three laboratories answered, listing one test each: FLT3; ABCB1; and ‘CGH [comparative genomic hybridisation] array NGS’.

### IP support

In the 2008 European survey, 37% of responding laboratories said that they did not have access to sufficient support to deal with patent-related issues, whereas 30% said they did have enough support (and 30% did not know). In the 2018 survey, more fine-grained answers to this question were sought and provided: 15% said that they never had support and 32% said they sometimes did not have support, whereas 5% said that always had sufficient support and 17% said they did most of the time (*n* = 156) (31% did not know). Although the questions were phrased differently, the results suggest that the availability of support has not improved.

### Legal policy implications

The central question motivating this study was whether Europe should follow the path of the US and restrict the patentability of gene-related patents. The legal mechanics for such a change are not straightforward [41], and evidence in support would need to be strong. Relevant evidence includes whether gene-related patents are having a negative impact on European clinical genetic laboratories.

Results from this study confirm that, to a degree, patents are posing challenges for European laboratories, and that the impact has increased since 2008. Most notably, the proportion of laboratories across the sector that have refrained from performing a test due to a patent has approximately doubled and the number of laboratories from non-profit organisations that have refrained has more than tripled (albeit both from a low baseline number) (Box 1). Furthermore, these impacts are observed across Europe (13 countries in our study), and are most pronounced in countries with large populations and high per-capita healthcare expenditure—i.e., the more-lucrative markets for commercial interests. The impact is also magnified by the marked increase in the number of clinical genetic testing laboratories. Where patents are having an impact, they are potentially affecting a greater number of organisations.

Five further results demonstrating the increased impacts or importance of patents on clinical genetic laboratories include: 16% more laboratories now knowingly conduct tests on patented genes or methods than a decade ago; 10% more now purchase kits that have licenses bundled into the purchase price; 28% of laboratories think the threat of patent enforcement is an important or very important reason to choose to use a commercial kit; almost one in five laboratories regard patents as an important or very important reason to outsource genetic tests; and a significant number of laboratories in specific countries have been contacted about patent infringement, namely: 100% (2/2) of the Swedish, 40% (2/5) of the Belgian and 32% (6/19) of the UK laboratories that participated in this survey.

These results, however, must be put in context. For example: 76% of laboratories have not been contacted regarding patent infringement; the confidence intervals on some differences between the 2008 results and this survey are quite wide; and only 5% of laboratories have negotiated stand-alone patent licenses. In addition, 31% do not know if they have IP support, only 7% have changed research interests owing to a patent, and 29% do not know if they conduct tests on patented genes or methods. These results indicate that for a significant proportion of laboratories, patent issues are not a primary concern. Furthermore, if one were to separate NIPT from the analysis (for example, on the grounds it is a platform technology and therefore warrants a different approach in patent law), the impact of patents on laboratories is even smaller.

The negative impacts for laboratories outlined above are not in and of themselves negative for patients and the healthcare system. For instance, the mere fact that a patent leads a laboratory to discontinue a test is a negative impact on that lab, but it is not necessarily negative for a healthcare system or patients. The impact on patients depends on several factors, for example: is there a satisfactory alternative medical pathway; can the patients obtain the test from a different laboratory; can funds be raised for a reasonable patent license; has the patent boosted follow-on innovation such that there is net social benefit?

Considered in the round, our view is that the results from this study do not justify European patent law reform at present. The 2017 expert review of the 98/44 EU Biotech Directive reached a similar conclusion with respect to isolated DNA sequence patents [42]. In our view, there is not enough evidence of a problem, particularly given the relatively low number of genetic tests affected. The US experience also shows that law reform in this area is challenging [43], and, especially a decision like *Mayo*, is likely to lead to undesirable uncertainty for those involved in translational research on new molecular tests [44–46].

That said, our survey shows that the patent system is likely to continue throwing up occasional problems as innovation develops. Over time, the current crop of problematic patents will expire and others will probably take their place. For instance, as the survey concluded, the foundational patent for NIPT has expired, as has the patent for FLT3. At any point in time there is likely to be a handful of patents resulting in blocking, licensing disagreements, or outsourcing of testing or research. European laboratories and their patients deserve some sort of support to help deal with these problems.

Box 1 Key results at a glance Compared with a decade ago:

- Laboratories that have refrained from offering a test due to a patent increased from 7 to 15%.
- Non-profit laboratories that refrained from offering test due to a patent increased from 4 to 14%.
- Laboratories that knowingly conducted tests on patented genes or methods increased from 22 to 37%

But impacts are still not wide spread. In 2017:

- 76% of laboratories have not been contacted about infringement.
- 7% have changed research interests due to a patent.

## Conclusion and recommendations

To help European laboratories manage the increased burden of gene-related patents, we recommend laboratories, particularly public sector laboratories, be given better IP legal support. This survey shows that although the impact of patents has increased in some respects, legal support has not improved over the same time-frame. This is despite repeated recommendations for better support over the past 15 years [5, 47].

Among other things, improved legal support would help laboratories: avoid high risks of patent infringement; identify ways to reduce the impact of patents (e.g., through licensing or outsourcing); and notify laboratories when key patents have expired or are no longer maintained in particular jurisdictions [48]. Useful general information could be organised and disseminated through general public channels (e.g., health departments, national patent offices, consultant reports or academic publications). Ideally, improved legal support would be tailored to specific organisations (e.g., university or research institute) and local conditions (e.g., country and tests offered). It is important that the advice is smart and not overly cautious; many granted patents have doubtful validity [49–52] and, in the right circumstances, ignoring patents can be the best strategy. As the IP scholar, Mark Lemley, notes: “[v]irtually everyone does it” [53]. Better IP support would enable laboratories to ignore patents more strategically.

Further consideration could be given to centralised-licensing of key patents by government bodies on behalf of public hospitals that wish to use the patents. This might help address concerns that stand-alone license fees are unreasonable. Any such efforts, though, should be careful not to delay unduly implementation of tests or lock laboratories into tests that may become obsolete.

We also recommend watchful monitoring of gene-related patent impacts. The trajectory over the past 10 years for European laboratories is concerning, and there are signs that negative impacts will continue, albeit fluctuating, for some time to come. For instance, our survey found that patent owners tend to enforce patents against large or for-profit laboratories, meanwhile the survey also found marked growth in these sectors of the industry. It is also significant that although the number of tests affected by gene-related patents is currently small, one is a platform technology (NIPT) with a multitude of applications. The primary companies controlling the technology (Illumina and Sequenom) [54], in part through their patent positions, have strong market positions that could cause widespread issues in the years ahead.

## Supplementary information

Supplementary Material
