# Supplementary material for: Continental drift? Do European clinical genetic testing laboratories have a patent problem?
Source: Eur J Hum Genet. 2019 Mar 7;27(7):997–1007. doi: 10.1038/s41431-019-0368-7 (PMC6777525; doi:10.1038/s41431-019-0368-7)
Supplement: Supplementary file 1 — Supplementary Material [file 41431_2019_368_MOESM1_ESM.docx]

Supplementary Material

Table of Contents

[A. Extra Results 2](#_Toc521601601)

[i. Does your laboratory perform tests in any of the following areas? Please select more than one if appropriate. 2](#_Toc521601602)

[ii. What type of molecular genetic testing does your laboratory perform? Please select more than one if appropriate. 2](#_Toc521601603)

[iii. What is your role in the laboratory? 3](#_Toc521601604)

[iv. Have any of the staff in your laboratory been involved in applying for patents on any aspect of molecular genetic testing? 3](#_Toc521601605)

[v. Tests that laboratories paying for that included bundled licences 3](#_Toc521601606)

[vi. Prominent reasons to *not* provide NIPT and FLT3 4](#_Toc521601607)

[a) FLT3 4](#_Toc521601608)

[b) NIPT 5](#_Toc521601609)

[B. Non-statistical associations (shown using cross-tabulations) 5](#_Toc521601610)

[i. Laboratories that conduct tests using patented genes or methods 5](#_Toc521601611)

[ii. Laboratories that have been contacted about alleged patent infringement 6](#_Toc521601612)

[iii. Laboratories that purchase kits with bundled licences 7](#_Toc521601613)

[iv. Laboratories that think the risk of patent enforcement is a very important or important consideration to buy a kit 7](#_Toc521601614)

[v. Laboratories that chose not to provide a test due to a patent 8](#_Toc521601615)

[C. Estimate of Labs that Received the Survey Email 8](#_Toc521601616)

[D. Confidence intervals 9](#_Toc521601617)

[i. Equation Used 9](#_Toc521601618)

[i. Proportion of laboratories from for-profit organisations, 2008 v 2017 9](#_Toc521601619)

[ii. Proportion of laboratories that conduct tests on patented genes or methods, 2008 v 2017 9](#_Toc521601620)

[iii. Proportion of laboratories that purchased kits with bundled licences 9](#_Toc521601621)

[iv. Proportion of laboratories that chose not to conduct a test due to a patent, 2008 v 2017 9](#_Toc521601622)

[v. Proportion of laboratories that pay licences fees or royalties, 2008 v 2017 10](#_Toc521601623)

[vi. Proportion of laboratories that provide <100 test and >5000, 2008 v 2017 10](#_Toc521601624)

[a) Analysis of laboratories that provide <100 tests 10](#_Toc521601625)

[b) Analysis of laboratories that provide >5000 tests 11](#_Toc521601626)

# Extra Results

## Does your laboratory perform tests in any of the following areas? Please select more than one if appropriate.

| # | Answer | % | Count |
| --- | --- | --- | --- |
| 1 | Genetic population screening | 3.92% | 27 |
| 2 | Pre-symptomatic screening | 12.05% | 83 |
| 3 | Prenatal | 15.24% | 105 |
| 4 | Newborn screening | 6.24% | 43 |
| 5 | Carrier testing | 18.87% | 130 |
| 6 | Susceptibility testing | 10.45% | 72 |
| 7 | Diagnostic testing to determine disease subtype/prognosis | 22.21% | 153 |
| 8 | Pharmacogenetics | 7.26% | 50 |
| 9 | I prefer not to answer | 0.00% | 0 |
| 10 | Other | 1.31% | 9 |
| 11 | Pre-implantation/IVF | 2.47% | 17 |
|  | Total | 100% | 689 |

Other: 1. Therapeutic; 2. Predictive markers testing and diagnostic markers; 3. Transplantation; 4. Cancer somatic mutations detection; 5. Nutrigenetics; 6. Follow-up and MRD; and 7. Virology.

## What type of molecular genetic testing does your laboratory perform? Please select more than one if appropriate.

| # | Answer | % | Count |
| --- | --- | --- | --- |
| 1 | Blood/tissue compatability | 2.95% | 16 |
| 2 | Cancer/Oncology | 22.14% | 120 |
| 3 | Neurological disorders | 18.27% | 99 |
| 4 | Infectious diseases | 5.17% | 28 |
| 5 | Blood disorders | 13.28% | 72 |
| 6 | Immunological disorders | 6.09% | 33 |
| 7 | Other inherited disorders | 23.99% | 130 |
| 8 | Other non-inherited disorders | 6.83% | 37 |
| 9 | I prefer not to answer | 0.00% | 0 |
| 10 | Other | 1.29% | 7 |
|  | Total | 100% | 542 |

Other: 1. Bone; 2. Somatic variants in tumour tissue; 3. Trisomy; 4. Imprinting diseases; 5. NIPT; 6. Hemostase (sub-group for blood)…; and 7. Congentital malformation.

## What is your role in the laboratory?

| # | Answer | % | Count |
| --- | --- | --- | --- |
| 1 | Laboratory director | 56.84% | 108 |
| 2 | Laboratory supervisor | 16.84% | 32 |
| 3 | Laboratory scientist | 13.16% | 25 |
| 4 | Marketing/administration staff | 1.05% | 2 |
| 6 | Intellectual property rights (IPR) expert | 0.53% | 1 |
| 8 | Clinician | 2.11% | 4 |
| 9 | Contracts manager | 0.00% | 0 |
| 7 | Other | 9.47% | 18 |
|  | Total | 100% | 190 |

Other: 1. Scientific assistant; 2. Head of Genetic Center; 3. Head of Dept; 4. Clinical molecular geneticist; 5. Analyst in laboratory medicine; 6. Laboratory technician; 7. Responsible for QA and development; 8. Clinical molecular geneticist; 9. Head of Dept; 10. Head of Dept; 11. Scientific director; 12. Head practise; 13. Quality manager; 14. Operational manager; 15. Scientific director; 16. Researcher. (two respondents who selected ‘other’ did not describe their position).

## Have any of the staff in your laboratory been involved in applying for patents on any aspect of molecular genetic testing?

| # | Answer | % | Count |
| --- | --- | --- | --- |
| 1 | Yes | 20.95% | 22 |
| 2 | No | 70.48% | 74 |
| 3 | I do not know | 8.57% | 9 |
|  | Total | 100% | 105 |

## Tests that laboratories are paying for that included bundled licences

1. Big Dye terminator kit, MLPA kit, BRCA multiplicon kit, Taqman Assay; 2. Tumor Hotspot from Multiplicom; 3. RIDA@ PRECISION ABCB1, R-Biopharm AG, An der neuen Bergstraße 17, D-64297 Darmstadt, Germany; 4. sequencing kits Thermofisher & illumine; 5. MRC Holland ... several MLPA kits; 6. Too much work, but the PCR license is included in most kits used, same for NGS; 7. AmplideX PCR/CE FMR1 Reagents (Asuragen), MLPA Kits (MRC-Holland); 8. QIAGEN; 9. IVS In-Vitro Scribe; 10. ELUCIGENE for CME testing. I am not sure about the MLPA kits of MRC-Holland; it is not the case for the kits from the Company MULTIPLICOM, I believe; 11. CFTR kit; Dyviser; 12. Amplidex, Assuragen; MLPA, MRC Holland; CFEU2, Elucigene.

## Prominent reasons to *not* provide NIPT and FLT3

Laboratories that chose not to provide a NIPT or FLT3 due to a patent were asked to rank six reasons why they chose not to perform a test. These were:

1. Unreasonable licensing or royalty cost;
2. Turn around time due to third party insistence that the test be completed elsewhere
3. Lack of confidence in the test due to third party insistence that the test be completed elsewhere
4. Third party will not provide or prevents my lab from obtaining reagents required for test
5. Risk of patent enforcement
6. Other

The optional rankings for respondents were:

1. Deciding factor
2. Very important consideration
3. Quite a relevant consideration
4. Marginally relevant consideration; and
5. Not a relevant consideration

To determine the most prominent reason, we calculated a weighted average rating for each reason. Weights assigned ranged from 4 for deciding factor through to 0 for not a relevant consideration. If a reason was ranked as a deciding factor, then it was given as a score of ‘4’ and if a reason ranked as a ‘very important consideration’ it was given a score of ‘1’. All other rankings were given a score of zero. This meant that the reasons with the highest scores were the most prominent. The results for both tests are below.

### FLT3

| **Reason** | **Number of Raters** | **Weighted Average Rating** |
| --- | --- | --- |
| Unreasonable licensing or royalty cost | 5 | 3.0 |
| Turn around time due to third party insistence that the test be completed elsewhere | 5 | 1.8 |
| Lack of confidence in the test due to third party insistence that the test be completed elsewhere | 5 | 1.8 |
| Third party will not provide or prevents my lab from obtaining reagents required for test | 5 | 1.8 |
| Risk of patent enforcement | 6 | 3.3 |

### NIPT

| **Reason** | **Number of Raters** | **Weighted Average Rating** |
| --- | --- | --- |
| Unreasonable licensing or royalty cost | 3 | 2.7 |
| Turn around time due to third party insistence that the test be completed elsewhere | 3 | 1.3 |
| Lack of confidence in the test due to third party insistence that the test be completed elsewhere | 3 | 1.3 |
| Third party will not provide or prevents my lab from obtaining reagents required for test | 3 | 2.7 |
| Risk of patent enforcement | 3 | 3.0 |

# Non-statistical associations (shown using cross-tabulations)

## Laboratories that conduct tests using patented genes or methods

|  | | **Do you undertake tests on patented genes or patented methods of genetic diagnosis?** | | | |
| --- | --- | --- | --- | --- | --- |
|  |  |  |  |  |  |
|  |  | **Yes** | **No** | **I do not know** | **Total** |
| **Approximately how many molecular genetic test reports does your laboratory issue to clients or patients per year?** | **<100** | 0 | 6 | 5 | **11** |
|  | **100-499** | 10 | 20 | 9 | **39** |
|  | **500-999** | 9 | 7 | 7 | **23** |
|  | **1000-5000** | 34 | 21 | 21 | **76** |
|  | **>5000** | 17 | 9 | 13 | **39** |
|  | **Total** | **70** | **63** | **55** | **188** |
|  |  |  |  |  |  |
| **Do you have the support and information you need to deal with patent-related issues?** | **Always** | 4 | 2 | 2 | **8** |
|  | **Most of the time** | 15 | 8 | 4 | **27** |
|  | **Sometimes** | 20 | 17 | 13 | **50** |
|  | **Never** | 9 | 8 | 6 | **23** |
|  | **I do not know** | 11 | 17 | 20 | **48** |
|  | **Total** | **59** | **52** | **45** | **156** |

## Laboratories that have been contacted about alleged patent infringement

|  | | **Has your laboratory been contacted by a patent holder regarding alleged patent infringement?** | | | |
| --- | --- | --- | --- | --- | --- |
|  |  |  |  |  |  |
|  |  | **Yes** | **No** | **I do not know** | **Total** |
| **Is your organisation ...?** | **for profit** | 6 | 24 | 8 | **38** |
|  | **non-profit** | 15 | 100 | 10 | **125** |
|  | **Total** | **21** | **124** | **18** | **163** |
|  |  |  |  |  |  |
| **What type of molecular genetic testing does your laboratory perform? Please select more than one if appropriate.** | **Blood/tissue compatability** | 2 | 10 | 1 | **13** |
|  | **Other** | 1 | 5 | 0 | **6** |
|  | **Cancer/Oncology** | 18 | 75 | 11 | **104** |
|  | **Neurological disorders** | 12 | 64 | 9 | **85** |
|  | **Infectious diseases** | 3 | 18 | 4 | **25** |
|  | **Blood disorders** | 14 | 41 | 7 | **62** |
|  | **Other inherited disorders** | 16 | 82 | 14 | **112** |
|  | **Other non-inherited disorders** | 7 | 19 | 4 | **30** |
|  | **I prefer not to answer** | 0 | 0 | 0 | **0** |
|  | **Immunological disorders** | 4 | 19 | 6 | **29** |
|  | **Total** | **21** | **126** | **18** | **165** |
|  |  |  |  |  |  |
| **Approximately how many molecular genetic test reports does your laboratory issue to clients or patients per year?** | **<100** | 0 | 9 | 0 | **9** |
|  | **100-499** | 0 | 31 | 3 | **34** |
|  | **500-999** | 3 | 17 | 0 | **20** |
|  | **1000-5000** | 10 | 47 | 8 | **65** |
|  | **>5000** | 8 | 22 | 7 | **37** |
|  | **Total** | **21** | **126** | **18** | **165** |
|  |  |  |  |  |  |
| **Do you undertake tests on patented genes or patented methods of genetic diagnosis?** | **Yes** | 14 | 42 | 6 | **62** |
|  | **No** | 4 | 49 | 3 | **56** |
|  | **I do not know** | 3 | 35 | 9 | **47** |
|  | **Total** | **21** | **126** | **18** | **165** |
|  |  |  |  |  |  |
| **Do you have the support and information you need to deal with patent-related issues?** | **Always** | 0 | 8 | 0 | **8** |
|  | **Most of the time** | 7 | 19 | 1 | **27** |
|  | **Sometimes** | 11 | 35 | 4 | **50** |
|  | **Never** | 2 | 18 | 3 | **23** |
|  | **I do not know** | 1 | 39 | 7 | **47** |
|  | **Total** | **21** | **119** | **15** | **155** |

## Laboratories that purchase kits with bundled licences

|  | | **To your knowledge, are licences/royalties for patents incorporated into any of the purchase prices for these kits?** | | | |
| --- | --- | --- | --- | --- | --- |
|  |  |  |  |  |  |
|  |  | **Yes** | **No** | **I do not know** | **Total** |
| **Do you undertake tests on patented genes or patented methods of genetic diagnosis?** | **Yes** | 17 | 5 | 37 | **59** |
|  | **No** | 0 | 6 | 39 | **45** |
|  | **I do not know** | 3 | 1 | 39 | **43** |
|  | **Total** | **20** | **12** | **115** | **147** |

## Laboratories that think the risk of patent enforcement is a very important or important consideration to buy a kit

|  |  | **Is your organisation ...?** | | |
| --- | --- | --- | --- | --- |
|  |  |  |  |  |
|  |  | **for profit** | **non-profit** | **Total** |
| **Generally speaking, please rate the importance of the following considerations in deciding to use a kit over a 'homebrew' test - Risk of patent enforcement** | **Very important** | 0 | 7 | **7** |
|  | **Important** | 17 | 15 | **32** |
|  | **Neutral** | 11 | 45 | **56** |
|  | **Unimportant** | 2 | 12 | **14** |
|  | **Very unimportant** | 2 | 3 | **5** |
|  | **Not a relevant consideration** | 3 | 24 | **27** |
|  | **Total** | **35** | **106** | **141** |

## Laboratories that chose not to provide a test due to a patent

|  | | **Has your laboratory chosen not to perform a molecular genetic test because of a patent or patent-related issues (e.g. patent position is too unclear, paying licence fee would set a problematic precedent)?** | | | |
| --- | --- | --- | --- | --- | --- |
|  |  |  |  |  |  |
|  |  | **Yes** | **No** | **I do not know** | **Total** |
| **Is your organisation ...?** | **for profit** | 8 | 22 | 9 | **39** |
|  | **non-profit** | 18 | 89 | 21 | **128** |
|  | **Total** | **26** | **111** | **30** | **167** |
|  |  |  |  |  |  |
| **Do you undertake tests on patented genes or patented methods of genetic diagnosis?** | **Yes** | 13 | 37 | 13 | **63** |
|  | **No** | 6 | 42 | 9 | **57** |
|  | **I do not know** | 7 | 34 | 8 | **49** |
|  | **Total** | **26** | **113** | **30** | **169** |
|  |  |  |  |  |  |
| **Has your laboratory been contacted by a patent holder regarding alleged patent infringement?** | **Yes** | 12 | 8 | 1 | **21** |
|  | **No** | 11 | 98 | 17 | **126** |
|  | **I do not know** | 0 | 7 | 11 | **18** |
|  | **Total** | **23** | **113** | **29** | **165** |
|  |  |  |  |  |  |
| **Do you have the support and information you need to deal with patent-related issues?** | **Always** | 1 | 5 | 2 | **8** |
|  | **Most of the time** | 7 | 17 | 3 | **27** |
|  | **Sometimes** | 13 | 31 | 6 | **50** |
|  | **Never** | 1 | 19 | 3 | **23** |
|  | **I do not know** | 1 | 36 | 11 | **48** |
|  | **Total** | **23** | **108** | **25** | **156** |

# Estimate of Labs that Received the Survey Email

The number of inactive email addresses on Orpha.net was estimated from a cohort of follow-up interviews. After the survey closed on 6 July 2017, 40 laboratories from each Germany and the UK that were listed only on Orpha.net were randomly selected for follow-up phone interviews. The laboratories were asked if they received the survey email. 38 of the 80 (47%) laboratories did *not* receive the email, usually because the email address listed on Orpha.net was no longer used. If this proportion is extrapolated to all the laboratories listed only on Orpha.net, this means that only approximately 245 of the 466 laboratories listed only on Orpha.net actually received the email. No means exist to determine whether the laboratories listed on both Orpha.net and EMQN received the email. Assuming all laboratories listed on both EQMN and Orpha.net received the email, the survey was sent to approximately 806 laboratories with active email addresses.

# Confidence intervals

## Equation Used

90% Confidence intervals were calculated using the ‘Wald confidence interval’ with continuity corrections:^[[1]](#endnote-1)^

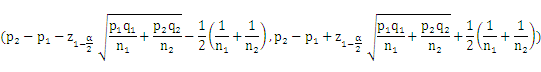


The equation was implemented using the Centre for Clinical Research and Biostatistics (Chinese University of Hong Kong) online calculator (available [here](https://www2.ccrb.cuhk.edu.hk/stat/confidence%20interval/CI%20for%202-proportions.htm)^[[2]](#endnote-2)^).

## Proportion of laboratories from for-profit organisations, 2008 v 2017

2008: n = 83, 6 were for-profit (7%)

2017: n = 188, 47 were for-profit (25%)

Difference between proportion of profit making laboratories (2008 v 2017) = 17.77%; 90% lower limit CI = 9.912%; 90% upper limit CI = 25.627%.

## Proportion of laboratories that conduct tests on patented genes or methods, 2008 v 2017

2008: n = 83, 18 (21.687%) conducted tests on patented genes or methods.

2017: n = 188, 70 (37.234%) conducted tests on patented genes or methods.

Difference between proportion of laboratories conducted tests on patented (2008 v 2017) = 15.55%; 90% lower limit CI = 5.245%; 90% upper limit CI = 25.849%

## Proportion of laboratories that purchased kits with bundled licences

2008: n=83, 3 (3.614%) purchased kits with bundled licences.

2017: n=147, 20 (13.605%) purchase kits with bundled licences.

Difference in proportion laboratories that purchased kits with bundled licences = 9.991%; 90% lower limit CI = 3.305%; 90% upper limit CI = 16.677%.

## Proportion of laboratories that chose not to provide a test due to a patent, 2008 v 2017

2008: n= 83, 6 (7.228%) chose not to provide a test.

2017: n= 169, 26 (15.385%) chose not to provide a test.

Difference in proportion of laboratories that chose not to provide a test due to a patent (2008 v 2017) = 8.157%; 90% lower limit CI = 0.724%; 90%; upper limit CI = 15.590%.

## Proportion of laboratories from non-profit organizations that chose not to provide a test due to a patent, 2008 v 2017

2008: n = 77, 3 (3.896%) chose not to provide a test.

2017: n = 18, 128 (14.063%) chose not to provide a test.

Difference in proportion of laboratories from non-profit organisation that chose not to provide a test due to a patent (2008 v 2017) = 10.167%; 90% lower limit CI = 2.898%; upper limit CI = 17.422%.

## Proportion of laboratories that pay licences fees or royalties, 2008 v 2017

2008: n= 83, 1 (1.205%) paid a licence fee or royalty

2017: n= 169, 9 (5.325) paid a licence fee or royalty

Difference in proportions of laboratories that purchase kits with bundled licences = 4.12%; 90% lower limit CI = – 0.2353%; 90% upper limit CI = 8.475%

## Proportion of laboratories that provide <100 test and >5000, 2008 v 2017

The raw results for this question in the two surveys is:

**2008**

| **Answer** | **Count** | **%** |
| --- | --- | --- |
| <100 | 9 | 10.84% |
| 100-499 | 18 | 21.69% |
| 500-999 | 15 | 18.07% |
| 1000-5000 | 32 | 38.55% |
| >5000 | 9 | 10.84% |
| **Total** | **83** | **100%** |

**2017**

| **Answer** | **Count** | **%** |
| --- | --- | --- |
| <100 | 11 | 5.82% |
| 100-499 | 39 | 20.63% |
| 500-999 | 23 | 12.17% |
| 1000-5000 | 77 | 40.74% |
| >5000 | 39 | 20.63% |
| **Total** | **189** | **100%** |

### Analysis of laboratories that provide <100 tests

Different in proportion of laboratories that provide <100 tests = -5.02%

90% lower limit CI = -12.16; 90% upper limit CI = 2.12%

### Analysis of laboratories that provide >5000 tests

Difference in proportion of laboratories that provide >5000 = 9.79%

90% lower limit CI = 1.51%; 90% upper limit CI = 18.06%

1. Fleiss, Levin and Paik, *Statistical Methods for Rates and Proportions* (Wiley-Interscience, 2003, 3^rd^) p 60; Fagerland, Lydersen and Laake. Recommended confidence intervals for two independent bionomical proportions. *Stat Methods Med Res* **24**, 224–254 (2015). [↑](#endnote-ref-1)
2. C.I. Calculator: Two Sample Proportions (Centre for Clinical Research and Biostatistics, The Chinese University of Hong Kong, Hong Kong) <https://www2.ccrb.cuhk.edu.hk/stat/confidence%20interval/CI%20for%202-proportions.htm>. [↑](#endnote-ref-2)
